# Directing three-dimensional multicellular morphogenesis by self-organization of vascular mesenchymal cells in hyaluronic acid hydrogels

**DOI:** 10.1186/s13036-017-0055-6

**Published:** 2017-04-03

**Authors:** Xiaolu Zhu, Shiva Gojgini, Ting-Hsuan Chen, Peng Fei, Siyan Dong, Chih-Ming Ho, Tatiana Segura

**Affiliations:** 1 College of Mechanical and Electrical Engineering, Hohai University, Changzhou, Jiangsu 213022 China; 2 Mechanical and Aerospace Engineering Department, University of California Los Angeles, Los Angeles, CA 90095 USA; 3 Chemical and Biomolecular Engineering Department, University of California Los Angeles, Los Angeles, CA 90095 USA; 4 Department of Mechanical and Biomedical Engineering, City University of Hong Kong, Hong Kong, China; 5 Bioengineering Department, University of California Los Angeles, Los Angeles, CA 90095 USA

**Keywords:** Self-organization, Hyaluronic acid, Turing mechanism, Mesenchymal stem cell

## Abstract

**Background:**

Physical scaffolds are useful for supporting cells to form three-dimensional (3D) tissue. However, it is non-trivial to develop a scheme that can robustly guide cells to self-organize into a tissue with the desired 3D spatial structures. To achieve this goal, the rational regulation of cellular self-organization in 3D extracellular matrix (ECM) such as hydrogel is needed.

**Results:**

In this study, we integrated the Turing reaction–diffusion mechanism with the self-organization process of cells and produced multicellular 3D structures with the desired configurations in a rational manner. By optimizing the components of the hydrogel and applying exogenous morphogens, a variety of multicellular 3D architectures composed of multipotent vascular mesenchymal cells (VMCs) were formed inside hyaluronic acid (HA) hydrogels. These 3D architectures could mimic the features of trabecular bones and multicellular nodules. Based on the Turing reaction–diffusion instability of morphogens and cells, a theoretical model was proposed to predict the variations observed in 3D multicellular structures in response to exogenous factors. It enabled the feasibility to obtain diverse types of 3D multicellular structures by addition of Noggin and/or BMP2.

**Conclusions:**

The morphological consistency between the simulation prediction and experimental results probably revealed a Turing-type mechanism underlying the 3D self-organization of VMCs in HA hydrogels. Our study has provided new ways to create a variety of self-organized 3D multicellular architectures for regenerating biomaterial and tissues in a Turing mechanism-based approach.

**Electronic supplementary material:**

The online version of this article (doi:10.1186/s13036-017-0055-6) contains supplementary material, which is available to authorized users.

## Background

In recent years, there has been increasing demand for reliable therapies to regenerate damaged tissues and organs [1–5]. Many cell-based methods have been proposed to regenerate the functions of living tissues and organs [4, 6–8]. Methods based on complex and intricate scaffolds [9, 10], cell sheet technology [11], spatial manipulation of living cells, or a cell-jet printer [12–14] are used to engineer tissues and organs. Generation of these artificial tissues typically requires engineering methods that can guide cells to grow in the desired spatial distribution and allow cells respond to exogenous physical and/or chemical perturbations. Hence, biological fabrication must accommodate engineered stimulations with cellular interactions and form the desired tissue pattern. Additionally, such attempts require complex implementation procedures and may cause immunogenic reactions during scaffold seeding and degradation [15] or influence cellular differentiation due to scaffold stiffness [16] and geometric cues [17]. To overcome these limitations, there has been increasing interest in the study of self-organizing properties and the innate regenerative capability of the tissue or organ [18–20], which could provide opportunities for recapitulating the organization of native tissue.

To recapitulate the self-organizational events, cells must be cultivated in a well-defined physiological environment that mimics the natural extracellular matrix (ECM). Currently, engineered three-dimensional (3D) hydrogel matrices [8, 21] provide a physiological context that closely mimics the natural ECM; therefore, events such as intercellular communication, cell-matrix interactions and cellular self-assembly can be recapitulated in defined 3D matrices [22, 23]. These 3D culture strategies have produced some multicellular structures, including spheroids consisting of tumor or stem cells [24], lumen formed by glandular cells [24], bronchioalveolar-like branching networks [25], optic-cup-like structures [4], tubular structures [26, 27], and even liver organ buds [19]. However, the emergence of these structural morphologies in complex biological systems often cannot be deterministically produced, due to an insufficient understanding of the mechanisms underlying tissue and organ formation via multicellular self-organization. To further guide cellular behaviors and multicellular organization, many specialized techniques have been incorporated into the engineered ECM system, such as micro-patterning of hydrogels [28], nano-patterning of delicate bioactive signals [29], and establishing chemokine gradients in 3D matrices [30]. These attempts are typically more laborious than those involving self-organization of cells in a simple 3D hydrogel system without external assistance.

The cellular self-organization paradigm has been extended into vertebrate cell types and fundamental physiological processes. Vascular mesenchymal cell (VMC) is a stem cell-like multipotent cell that exhibits several remarkable capabilities for multicellular structure self-formation, which has been studied in our previous work [31, 32] and others’ work [33, 34]. Molecular regulation of the cell-to-cell and cell-to-ECM interaction plays crucial roles for development of 3D multicellular structures. Bone morphogenic protein (BMP, a member of transforming growth factor‑β super family), matrix GLA protein (MGP), and Noggin can serve as morphogenic signaling molecules [35–38]. In the self-organization paradigm based on a Turing’s mechanism [33, 34, 39], BMP and MGP act as a pair of activator and inhibitor during the reaction and diffusion of morphogens. Noggin proteins could serve as a signaling molecule, which may regulate the reaction/diffusion process of morphogens.

Here, we investigated control methods for VMCs to form desired self-organized patterns in 3D hydrogels. First, we experimentally demonstrated that the self-organization of VMCs formed 3D patterns in a modified hyaluronic acid (HA) hydrogel. Then, various 3D architectures were successfully developed in the VMC-HA hydrogel system by adjusting the components of the hydrogel and adding BMP2 and/or Noggin. These structures could mimic 3D patterns of trabecular bone or serve as a basic building block for creating complex 3D tissues [40]. In 2D studies, the Turing instability-governed reaction–diffusion process has been shown to be the probable mechanism underlying the 2D pattern formation of multipotent VMCs [31, 32, 34]. In this study, Turing instability can model engineered 3D multicellular architectures and approximately determine the transition point between different regimes of 3D multicellular morphology. The application of exogenous morphogens for development of 3D multicellular structures provides further chances for regulating the topology of 3D multicellular structures.

## Methods

### Cell culture

Vascular mesenchymal cells (VMCs) were isolated and cultured as previously described [41–43]. The cells were grown in Dulbecco’s Modified Eagle’s Medium (DMEM) supplemented with 15% heat-inactivated fetal bovine serum (FBS) and 1% penicillin/streptomycin (10,000 IU/10,000 μg/mL, Mediatech, VA). Cells were incubated at 37 °C in a humidified incubator (5% CO_2_ and 95% air). Cells were passaged every 3 days with 0.25% trypsin-EDTA following the standard protocol [31, 32, 43].

### Hyaluronic acid modification

Acrylated hyaluronic acid (HA-AC) was prepared using a two-step synthesis process [44, 45]. First, hyaluronic acid (2.0 g, 60 kDa, 5.28 mmol carboxylic acids, Genzyme Corporation, Cambridge, MA) was reacted with 36.8 g (211.1 mmol) of adipic dihydrazide (ADH) at pH 4.75 in the presence of 4.0 g (20 mmol) of 1-ethyl-3-[3-dimethylaminopropyl] carbodiimide hydrochloride (EDC) for overnight. The resulted HA-ADH was purified through dialysis (8000 MWCO) in deionized (DI) water for 2 days, lyophilized, and stored at −20 °C. Second, 1.9 g of HA-ADH was re-suspended in 4-(2-hydroxyethyl)-1-piperazine ethane-sulfonic acid (HEPES) buffer (10 mM HEPES, 150 mM NaCl, 10 mM EDTA, pH 7.4) and reacted with 1.33 g (4.4 mmol) of N-acryloxysuccinimide (NHS-AC) for overnight and purified through dialysis in DI water for 2 days before lyophilization. The final product HA-AC was analyzed with ^1^H NMR (D_2_O) and the degree of acrylation (16%) was determined by dividing the multiplet peak at δ = 6.2, which corresponds to cis and trans acrylate hydrogens, by the singlet peak at δ = 1.6, which corresponds to acetylmethyl protons in HA.

### VMC-laden HA Hydrogel formation

HA hydrogels were formed by the Michael Addition of bis-cysteine-containing MMP-degradable cross-linker (GCRDGPQGIWGQDRCG) onto HA-AC pre-functionalized with the Ac-GCGYG-RGDSPG-NH_2_ adhesion peptide (RGD, Genscript, Piscataway, NJ). Eight mg of lyophilized HA-AC was dissolved in 100 μL of 0.3 M TEOA buffer, mixed with the RGD peptides (0.1 mg/vial) in TEOA buffer, and placed for 25 min at 37 °C for production of HA-RGD. Single cell suspension was prepared and placed on ice. 1.0 mg of lyophilized cross-linker was then dissolved in 20 μL of 0.3 M TEOA buffer (pH = 8.4) and mixed with HA-RGD (final concentration of 100–150 μM RGD) and cell suspension. The gel precursor solution was pipetted drop-wise (10 μL per drop) onto a sigmacoted glass slide, then clamped with another sigmacoted slide with plastic cover slip spacers, and finally incubated for 30 min at 37 °C to allow for gelation. The final gel was swelled in culture medium before being placed inside 96-well plates for long-term culture. The mechanical properties of the hydrogels were controlled by varying the HA concentration, the crosslinking density, and “*r* ratio”, which is defined as the mole ratio of thiol (−SH) in the cross-linkers to acrylate group (−ACs) in the HA-ACs.

### Fixation and fluorescent staining

VMCs at initial cell density of 7,500/μL were seeded in the modified HA hydrogel. After treatment and long-term culture, the gel samples were washed twice in the micro-well plate with pre-warmed phosphate-buffered saline (PBS). Then, the cells were fixed by 4% paraformaldehyde (PFA) solution for 40 min at room temperature and were stained for 25 min at room temperature with Alexa Fluor® 488 phalloidin (Invitrogen) in PBS with 1% bovine serum albumin (BSA) for F-actins. Cell nuclei were stained with 300 nM DAPI (Invitrogen) in PBS for 5 min.

### Three-dimensional visualization with selective plane illumination microscopy (SPIM)

The HA gel samples stained by phalloidin and DAPI were immersed into an agarose solution (gelling point <30 °C) with a concentration of 0.5% (w/w) in PBS and were then placed in a transparent cuboid cuvette (Plastibrand, Germany). The agarose was gelled at room temperature and the HA gel samples were mounted inside the agarose gel. Then, the cuvette was placed onto the automatic stage of the SPIM platform. The sample was optically sliced at 1 or 2 μm. Images were acquired with SPIM imaging platform with 4x or 10x objectives, which typically provides a higher imaging quality than confocal microscopy under the same condition. The obtained image sequences were stacked, converted into binary images via thresholding, and processed by commercial 3D reconstruction software for 3D volume visualization (Amira 5.2 Resolve RT, trial version). The basic principle of SPIM was described in the Additional file 1: Supplementary Methods section and Additional file 2: Figure S1. The number of white voxels within the 3D volume was counted and the volume ratio of the multicellular structure to the 3D was calculated to characterize the degree of density of multicellular structures using an image process method that converted each grayscale image to binary by thresholding. The output binary images has values of 1 (white) for all camera pixels in the input image with luminance greater than thresholding level and 0 (black) for all other pixels. The number of white voxels was counted on each frame of the imaging sequence, and then the number of white voxels was summarized for all the image sequences. Cell occupied volume fraction (COVF) was characterized by the number ratio of white voxels to total voxels in the entire 3D regions.

### Rheology measurement for characterizing the stiffness of hydrogels

The storage (G’) and loss modulus (G”) were measured with a plate-to-plate rheometer (Physica MCR, Anton Paar, Ashland, VA) using an 8-mm plate under a constant strain of 0.05 and a frequency ranging from 0.1 to 10 rad/s. The hydrogels were cut to a size of 8.0 mm in diameter. A humid hood at 25 °C was used to prevent the hydrogel from drying.

## Results

### 3D pattern formation of VMCs in modified HA hydrogel

**Fig. 1:**
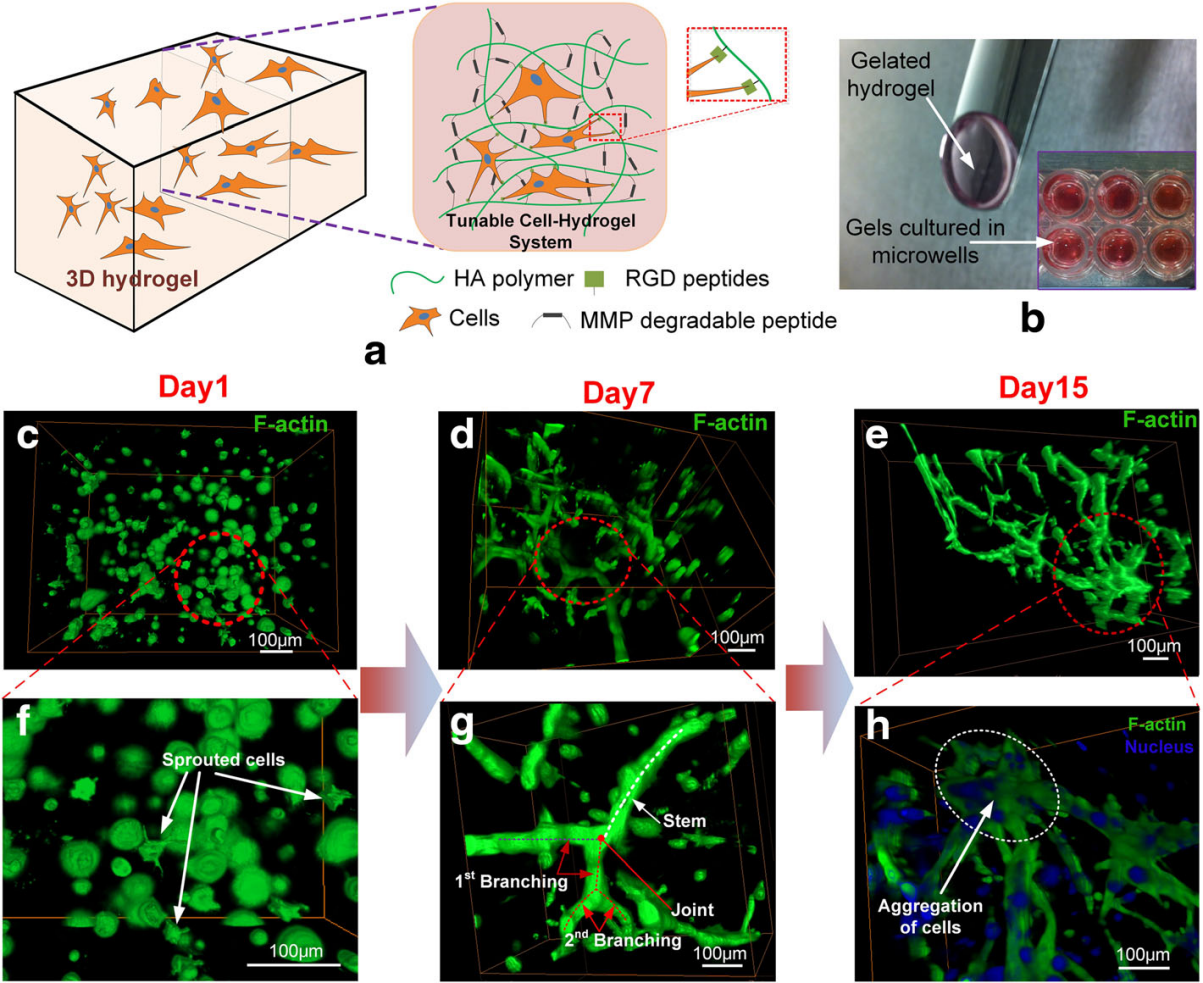
VMC pattern formation in 3D HA hydrogel. **a** Schematic presentation for 3D cell culture inside a modified semi-synthetic HA hydrogel. **b** Culture of gelated HA hydrogel-encapsulated cells in microwells. **c**–**h** Sprouting and branching of VMCs in HA hydrogel. In 3D culture, VMCs were distributed nearly uniformly in HA hydrogel at Day 1 (24 h after seeded) and few cells had sprouted **c**, **f**. On day 7, VMC cells formed a network with branched structures **d**, **g**. On day 15, VMC cells aggregated into thicker and bulkier structures **e**, **h**. VMC cell F-actin and nuclei were stained with phalloidin (green) and DAPI (blue). Scale bars 100 μm

The carboxyl groups in the HA backbone were modified with acrylate groups to incorporate RGD peptides, introduce integrin binding sites, crosslink the HA polymers, and generate a mechanically stable hydrogel through Michael addition chemistry. VMCs were encapsulated inside the modified HA hydrogel and the VMC cells attached to the RGD peptides were dispersed throughout a 3D space after the hydrogel gelled (Fig. 1a). Then, the HA hydrogel-encapsulated cells were cultured in micro-wells in cell culture plates (Fig. 1b). Both the onset and extent of cell spreading were influenced by RGD concentrations (Additional file 2: Figure S2a and b). Generally, cells spread earlier and migrated faster at higher concentrations of RGD; however, these cells had low proliferation rates. Moreover, cell behaviors were affected by the hydrogel stiffness that are determined by the HA concentration and cross linker density. The variation of morphology, migration and cell proliferation VMC under different RGD or crosslinker concentrations were presented in the supplementary materials (Additional file 2: Figure S2), which has the similar trend with the laws in previous literature [46, 47]. The components in this semi-synthetic HA hydrogel were optimized to provide an effective platform to study multicellular structure formation in a 3D matrix. In this study, the optimal conditions determined for multicellular structure formation were the HA hydrogels with 3.0–3.5% HA, 100–150 μM RGD and 0.4 – 0.75 thiol-to-acrylate ratio (r ratio).

The VMC, a stem cell-like multipotent cell that exhibit several remarkable capabilities for multicellular structure formation, has been studied in our Lab [31, 32] and in others [33, 34]. In our experiments, VMCs were distributed uniformly at beginning, but spread, migrated and proliferated inside the modified HA hydrogel network as a function of time. One day after seeding, VMCs were round and distributed randomly inside the 3D hydrogel (Fig. 1c). A few cells had small sprouting projections (Fig. 1f). After 2–3 days, the VMCs spread out with finger-like projections and started to interact with neighboring cells (Additional file 2: Figure S3a). The network of cells became more pronounced between day 4 and 8 (Additional file 2: Figure S3b–d). 3D multicellular networks that consisted of a large number of branches and sub-branches, formed by local multicellular aggregation at day 7 (Fig. 1d). Three features, stem, branches, and sub-branches, seemed to emerge at the same time. VMCs aggregated in local regions and then connected with each other under the combined stimuli of various physical and chemical signals in three dimensions. In the branching process, there was no specific order in which the stem, branches and sub-branches appeared (Fig. 1g). On day 15, local cells aggregated into a thicker and bulkier structure, as shown in Fig. 1e and h. As a function of time, VMCs finally formed a larger multicellular network that has a feature size of more than 200 μm (Fig. 1h) and is considerably larger than the structure composed of single branches as presented in Fig. 1d and g. After day 15, the morphology of the multicellular structure displayed no significant variation because VMC proliferation was hindered by limited space of HA hydrogel.

### Morphologic alterations of 3D multicellular structures by Noggin

**Fig. 2:**
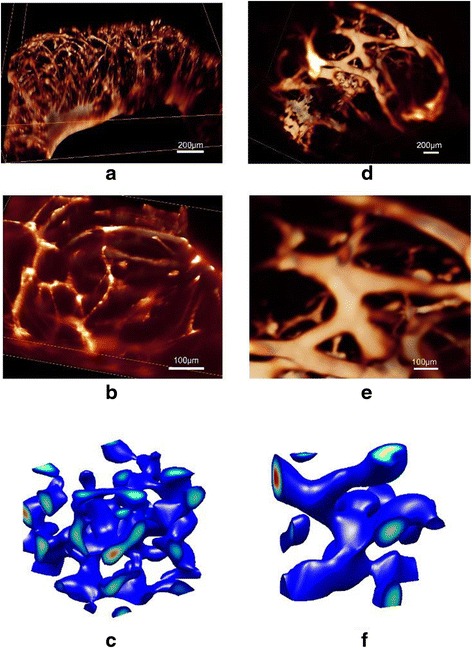
Alterations of the 3D self-organized VMC multicellular structure by Noggin. The multicellular structures were composed of aggregates of VMCs from thin networks (**a**) to thicker networks (**d**) (Day 11). **a**, **b** showed the experimental 3D multicellular structure without exogenous factors. **d**, **e** showed the experimental 3D multicellular structure treated by Noggin that was increased gradually from 0.25 to 0.9 μg/mL over the first 6 days and maintained at 0.9 μg/mL for the following days. **b** and (**e**) represented higher magnifications of (**a**) and (**d**), respectively. The multicellular structures were visualized by SPIM after F-actin staining with Alexa Fluor 488 phalloidin, which outlines the cellular skeletons. The conditions for VMC-HA hydrogel were 3.5% HA, 100 μM RGD, and initial cell density of 7,500/μL. **c** and (**f**) were the corresponding simulated 3D morphologies for (**b**) and (**e**), respectively. Regions of high cell concentrations were marked in red. The computational simulations showed the conversion from (**c**) a thin network structure (*D* = 0.005, *q* = 0.004, *χ* = 0.09, *U*_EX_ = 0, *V*_EX_ = 0), to (**f**) an aggregated thick network structure (*D* = 0.005, *q* = 0.006, *χ* = 0.09, *a* = 0.001, *U*_EX_ = 0, *V*_EX_ = 0)

An optimized hydrogel-VMC system (3.5% HA, 100 μM RGD, r = 0.5, initial cell density = 7,500/μL) was utilized to study the effect of Noggin on the 3D morphology of multicellular structures. In the control sample with approximately 1 million cells (Fig. 2a-b), the network structure formed upon VMC aggregation and connection with each other, but left hollow spaces between the connecting links. In the treated sample, Noggin was distributed uniformly when added to the cell-hydrogel system, to which VMCs responded significantly. Although Noggin initially influenced the biophysical condition of individual cell equally, the kinetics of VMC responses in different regions became distinct according to the observed evolution of multicellular morphology. The distribution of Noggin became non-uniform after the non-uniform 3D multicellular structure emerged because Noggin was consumed in a spatially non-uniform manner, determined by the spatiotemporal distribution of activators. After application of Noggin (0.25 to 0.9 μg/mL over the first 6 days and 0.9 μg/mL for the following days) to the 3D matrix, a different type of pattern, in which cells aggregated with structures of thick network geometry, was observed (Fig. 2d). This structure has locally thick aggregates of VMCs and branched/connected branches. Evolution of its morphology over time was shown in Additional file 2: Figure S4. A magnified image for the thick network of multicellular structure was presented in Fig. 2e. The large branch structure had dimensions of more than 100 μm. The crossed-multicellular limbs had largely arbitrary elongation orientations in the 3D matrix. We have therefore demonstrated that the morphologies of 3D multicellular structures can be modulated by exogenous morphogens such as Noggin within the predefined structural configuration of the hydrogel.

The observed morphologies of 3D multicellular architectures are most likely associated with a Turing-type mechanism [48, 49]. As first proposed by Turing [39], biological patterns relate to the spatial distributions of morphogens. Cellular patterns emerge from the instability caused by the reaction and diffusion of morphogens. In the reaction–diffusion framework, molecules produced by cells interact as activators and inhibitors (morphogen pairs) and diffuse through the ECM at different rates. Instability analyses of reaction–diffusion equations that model morphogen pairs, have been used to study pattern formation in many chemical and biological systems [34, 49–51].

BMP-2 and BMP-4, members of the TGF superfamily, and their inhibitor, matrix γ-carboxyglutamic acid protein (MGP), have been identified as morphogens secreted by VMCs [34, 52]. Here, we modeled the cell-hydrogel system as a slowly diffusing activator with an autocatalytic reaction that follows Gierer and Meinhardt kinetics and a rapidly diffusing inhibitor considering cytokinetic motility and chemotactic migration with respect to activators and using cell density to reflect proliferation as a function of a 3D domain (*x*, *y, z*) in dimensionless form:

1 $$ \frac{\partial U}{\partial t}= D{\nabla}^2 U+\gamma \left[\frac{pn{U}^2}{V\left(1+ k{U}^2\right)}- cU\right]- a U+{U}_{EX} $$

2 $$ \frac{\partial V}{\partial t}={\nabla}^2 V+\gamma \left[ bn{U}^2- eV\right]+{V}_{EX} $$

3 $$ \frac{\partial n}{\partial t}= q{\nabla}^2 n-\chi \left[\nabla \cdot \left(\frac{n}{{\left({k}_n+ U\right)}^2}\nabla U\right)\right]+{r}_n n\left(1- n\right) $$

In Eqs. (1, 2, 3), *U*, *V*, and *n* are dimensionless concentrations of activator, inhibitor, and cells, respectively, as functions of space coordinate (x, y, z) and time (*t*). *D*∇^2^ *U*, ∇^2^ *V* and *q*∇^2^ *n* are terms used to describe the diffusion of morphogens and cell. *c* and *e* are the degradation rates of the activator and inhibitor, and *b* is the coefficient representing the relative production of inhibitor to activator. *χ* is the regulation factor for chemotactic migration in response to the gradient of activators. *D* and *q* are the ratios of diffusion coefficients for activator to inhibitor and cells to inhibitor, respectively. *γ* is a scaling factor related to domain size, biosynthetic time-scale, and inhibitor diffusivity. The term − *aU* indicates the reduction of local concentration of activators when exogenous proteins bind and inactivate them. The coefficient *a* is dependent on the concentration of exogenous protein and efficiency of protein binding. *U*_*EX*_ and *V*_*EX*_ are the exogenous source terms for activator and inhibitor, respectively.

Simulation results for the experimental data in Fig. 2b and e were shown in Fig. 2c and f. Monomeric Noggin (50 kDa) has a slower diffusion rate than BMP-2 and BMP-4 [53]. Thus, Noggin does not satisfy the criteria that the inhibitor must diffuse more rapidly than the activator in Turing-type activator-inhibitor models. However, Noggin still regulates the inhibitory process of MGP on BMP-2/4 because Noggin binds BMP-4 with high affinity and subsequently abolishes BMP-4 activity [54]. Therefore, the activators will be non-uniformly inhibited because local regions with a larger concentration of *U* will have more activators inhibited, assuming the amount of ﻿Noggin﻿ is enough, as described as − *aU* in Eq. (1) (here *a* =1 × 10^−3^). This effect decreases ∇*U* by values on the order of 10^2^ at most of the positions in this 3D simulation space. The term − *aU* also reduces activator activity and makes *n*/(*k*_*n*_ + *U*)^2^ increase by values on the order of 0.1-10 in 3D space. Therefore, the product of *n*/(*k*_*n*_ + *U*)^2^ and ∇*U* decreases. Consequently, the chemotactic effect might be weakened and the distribution of cells was more scattered, resulting in a larger feature size of network structure. Based on the migration tests for cell clusters inside HA hydrogels with the above experimental conditions (Additional file 2: Figure S6), the average migration rate of VMCs in the Noggin-treated sample was greater than that in the control, therefore the parameter *q* in simmulatiion models were given values of 0.006 and 0.004 respectively for Noggin-treated and control ones. Because a larger *q* leads to a more rapid migration away from an aggregated cellular cluster, the larger feature sizes in the Noggin-treated sample are reasonable.

### Morphological changes of 3D multicellular structures by BMP2

**Fig. 3:**
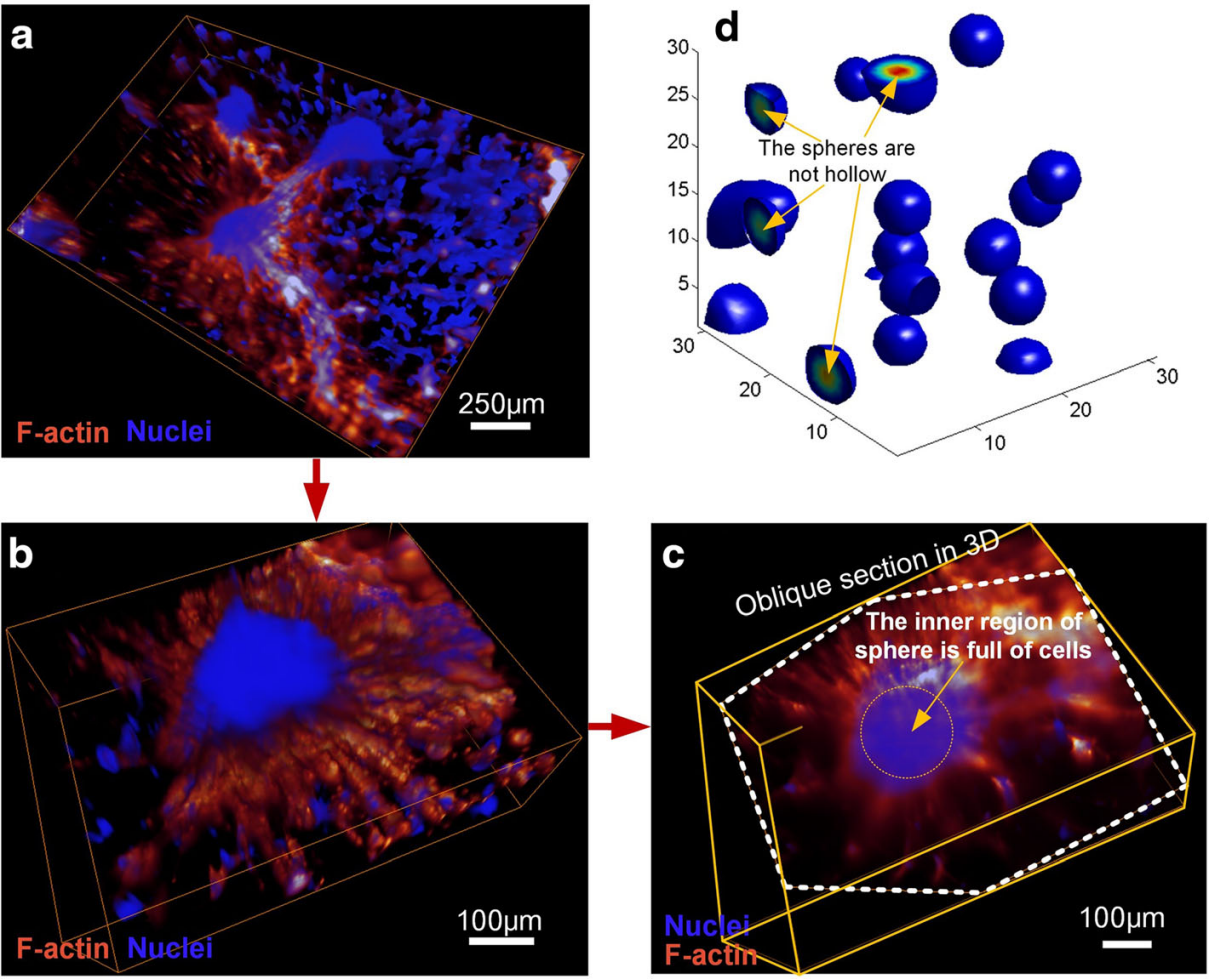
Self-organized 3D spheroids upon BMP2 treatment. **a** 3D VMC-HA hydrogel spheroids after 13-day stimulation with 500 ng/mL of BMP-2, the activator of MGP. **b** 2.5x magnification of (**a**). (**c**) Landscape after removal of the volume above the oblique section plane. **d** Simulation of a similar 3D morphology of distributed spheroids (*D* = 0.005, *q* = 0.003, *χ* = 0.10, *U*_EX_ = 8 × 10^−6^, *V*_EX_ = 0). Regions of high cell concentration were shown in red. F-actin outlining the cellular skeletons was stained with Alexa Fluor 488 phalloidin and VMC nuclei were stained with DAPI

When VMCs were stimulated with BMP-2, the activator of MGP, at a concentration of 500 ng/mL, 3D spheroids with diameters of approximately 200 μm formed 13 days later (Fig. 3a and b). Approximately one dozen spheroids, each packed with thousands of VMCs, were present inside each of the micro-wells of the hydrogel-based culture plate. Most of the spheroids were connected with each other (Fig. 3a) and spheroid formation process was shown in Additional file 2: Figure S5. The inner region of the spheroids was entirely composed of cells (Fig. 3c), which presented the landscape after removal of the volume above the oblique section plane. The mathematical model further predicted that addition of activator could convert the network structure to spheroids (Fig. 3d) because BMP-2 repressed the diffusive migration of cells (Additional file 2: Figure S6) and could stimulate the chemotactic migration [55]. In the model, the value of *q* was decreased from 0.004 to 0.003, the value of *χ* was increased from 0.09 to 0.10, and the exogenous source term *U*_*EX*_ took on a positive value of 8 × 10^−6^ to match the experimental results. The 3D spheres had no cavity inside (Fig. 3c), which was in consistent with simulation result (Fig. 3d) using the above parameter values.

**Fig. 4:**
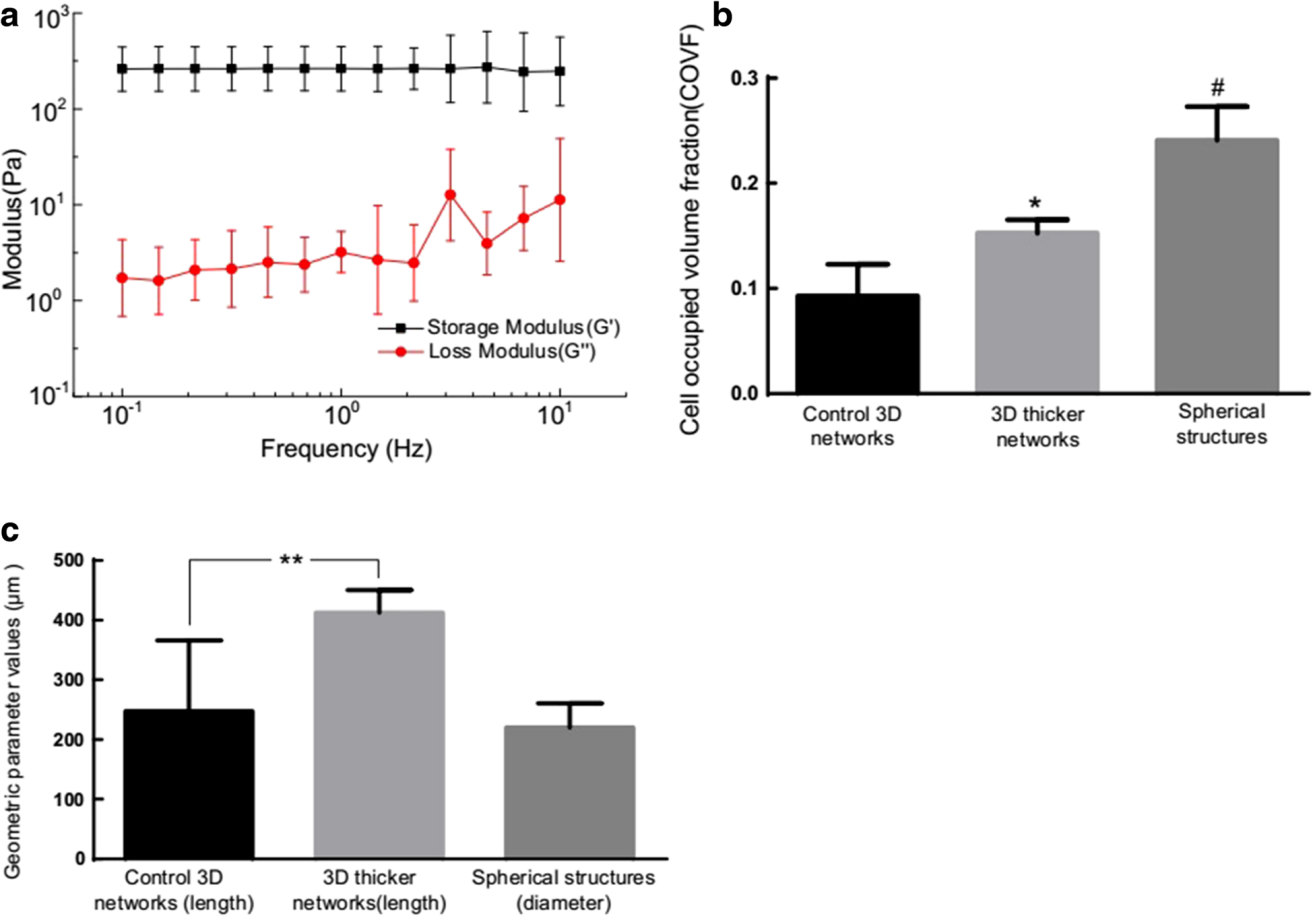
**a** The mechanical property of the HA hydrogel used in this experiment. **b** The volume ratio of the multicellular structure to the 3D matrix for the three types of multicellular structures. The cell occupied volume fractions (COVF, mean ± SD) for the three types of multicellular structures had statistically significant difference between each other. ^*^ *P* < 0.03 versus the control 3D networks, *n* = 4; ^#^ *P* < 0.001 versus the control 3D networks, *n* = 4. **c** Geometric parameter values including the characteristic length of network and the diameter of multicellular spheroid for three experimental groups. The characteristic lengths (mean ± SD) between adjacent network nodes had statistically significant difference in control and 3D thicker network samples. ^**^ *P* < 0.05; *n* =20

The mechanical property of the HA hydrogel used to generate these multicellular structures was characterized as shown in Fig. 4a. The storage (G’) and loss modulus (G”) did not cross at any measured frequency (0.1–10 Hz) and frequency independence were consistent with typical hydrogel characteristics. The loss tangent values (ratio of G” to G’) were lower than 0.05, indicating that the hydrogels were highly elastic. The dense degree of each type of multicellular structure could be characterized by the volume ratio of multicellular structure to the entire matrix 3D space (or denoted as cell occupied volume fraction, COVF) by image process methods. As indicated in Fig. 4b, the thin network structure (control) had a lowest COVF value (around 10%) while the aggregated 3D thicker network structure and spherical structure had higher COVF values indicating denser features of the multicellular structures. The characteristic lengths between adjacent network nodes could be used to characterizing the geometric features, and this “length” had statistically significant difference in control and 3D thicker network samples (Fig. 4c). The diameter of multicellular spheroid was also measured as a geometric parameter value that was between 150 μm and 280 μm (Fig. 4c).

### Combined effects of Noggin and BMP2 on 3D multicellular structures

**Fig. 5:**
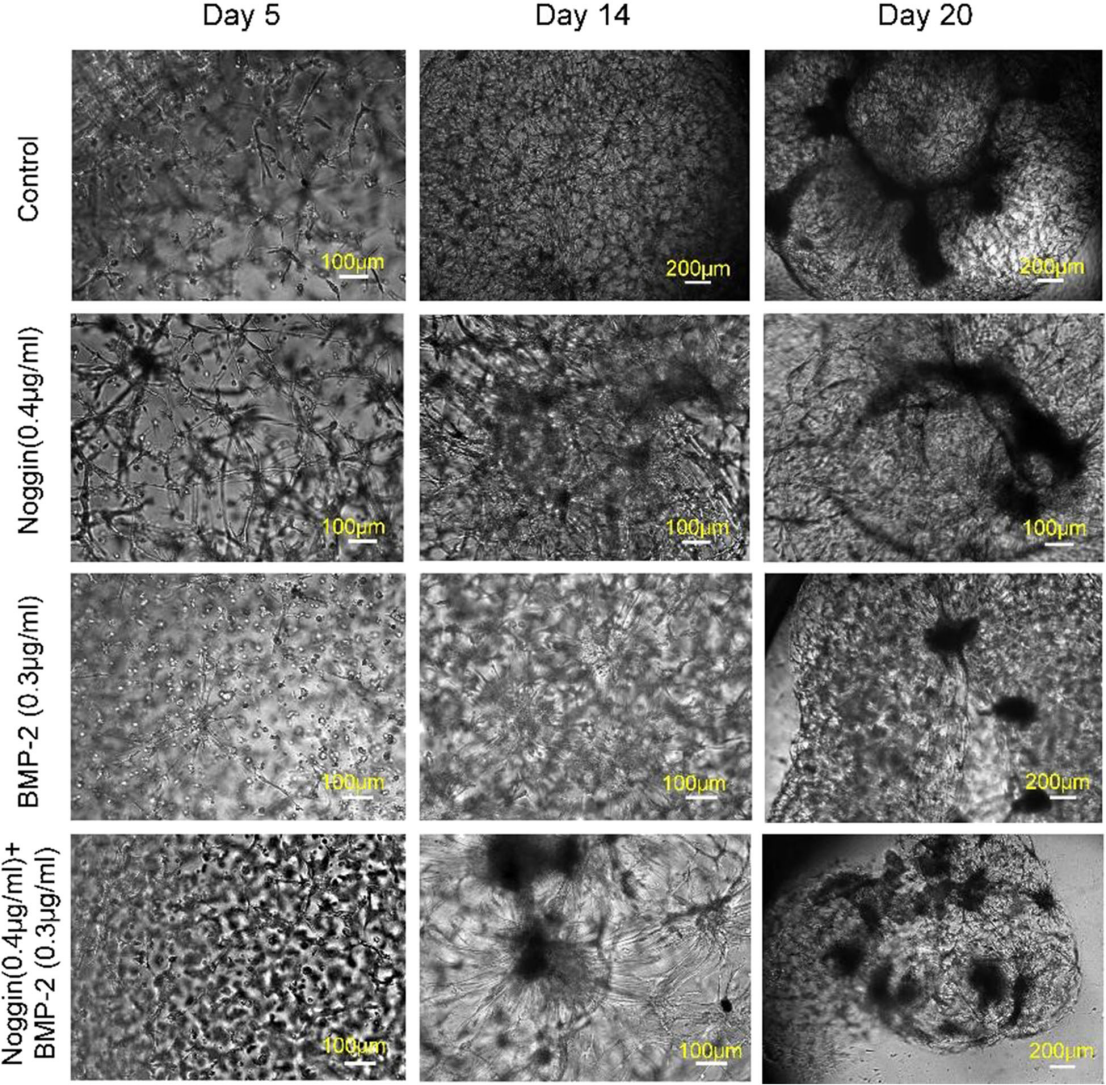
Effects of Noggin and BMP2 on VMC multicellular morphology. The experimental conditions for VMC-HA hydrogel system were 3.5% HA, 100 μM RGD, and *r* = 0.55

As discussed above, three different types of 3D multicellular architectures can be created by adding Noggin or BMP2. We next investigated combined effects of Noggin and BMP2 on VMC multicellular structures. VMCs were treated with 0.4 μg/mL Noggin and/or 0.3 μg/mL BMP2 and morphological changes were recorded at different culture times. When only BMP-2 was applied, VMCs formed spheroids (Fig. 5). More and larger spheroids emerged with longer culture duration. When only Noggin was applied, VMCs typically formed local aggregates and then formed small protrusions with irregular geometries. Those small protrusions became larger and denser after day 14. The combination of BMP-2 and Noggin led to complex multicellular morphologies in which miscellaneous morphological features coexisted. The observed morphologies upon treatment with Noggin and BMP2 presented irregular aggregation of cells at day 14. The geometry, size, and density of the aggregations varied from days 14 to 20. Finally, these multicellular structures grew further and had distinct, more aggregated, and complex topologies. Mapping the combined effects of Noggin and BMP2 could lay a foundation for promoting the capability and flexibility of the creation of multicellular structures with numerous different morphologies.

## Discussion

Our study has demonstrated that various 3D VMC cellular structures can be obtained by applying Noggin and BMP2 at specific concentrations. Turing instability mechanism models the 3D cell patterns well. Most of VMCs inside the 3D matrix differentiated into bone cells after 15 days (Additional file 2: Figure S7). Exogenous morphogens regulated the spatial characteristics and feature sizes of the remodeled trabecular bone structure. The regenerated sponge-like cellular structure could serve to generate diversified building blocks for vertebrae and large bones, such as the femur, tibia, and humerus in the human body. Utilizing the more physiologically relevant 3D multicellular spheroids rather than dissociated cells from 2D monolayer culture has the potential to enhance the VMCs’ survival and proangiogenic potential upon implantation into a bone defect site in clinical practice [56]. Moreover, multicellular aggregated spheroids can serve as a precondition for forming multicellular structure with branched tubular morphology [27]. The theoretical interpretations for 3D multicellular networks may provide possible approaches for understanding the mechanism of the age-related changes in trabecular architectures [57], for the self-organization of VMCs in bone tissue formation within artery walls that results in atherosclerotic lesions [34], and for the formation of 3D spheroids of aggregated cells that recapitulate the morphological and biochemical characteristics of embryonic/woven bone formed in vivo.

HA possesses many attractive qualities, including biocompatibility and biodegradability, and facilitates cells adhesion, proliferation, migration, and morphogenesis through its two surface receptors, CD44 and CD168 [58]. Hyaluronan also forms a multivalent template for interactions with proteoglycans and other extracellular macromolecules that is important in the assembly of extracellular and pericellular matrices[59]. These properties of HA help to regulate the porosity and malleability of these matrices, which are important factors in determining whether cells invade tissues during development, tissue remodeling, and cancer progression.

At the cellular level, HA is progressively degraded by a series of enzymatic reactions, generating polymers with decreasing sizes [60]. The degraded fragments of HA may modulate effects of the HA receptors, through size-specifically binding to hyaladherins [60] and stimulating angiogenesis in vivo [61, 62]. However, exogenous addition of small-sized HA molecules may not mimic effects of HA fragments generated in situ. Therefore, this study mainly relied on the degradation of HA by MMP and Hyaluronidase secreted by VMCs for generating HA fragments that could benefit to the implantation of the generated tissue precursors (hydrogel encapsulating multicellular structures) into the tissue defect site for angiogenesis in clinical practice.

Developing 3D multicellular structures in the tunable biological reaction–diffusion system provides a foundation for the rational design of 3D morphologies of tissue models and a controlled platform for studying the biological and engineering principles underlying spatiotemporal morphogenesis. Our theoretical model can predict the variation tendency of 3D morphology of multicellular structures, although the parameter values may not directly correspond to physical quantity value in experiments. Three distinct growth modes have been identified in our simulations, and they could serve as the guidance for continuous and discrete modulations in concentration of exogenous cytokines or other adjustable quantities, in order to deterministically switch between different regimes and create a bouquet of self-organized multicellular structures. Recently, we also experimentally created hollow tubular structures via self-organization of VMCs [63], which could be modeled under the Turing's reaction-diffusion frame, but still needs more insight to the underlying mechanism. The self-organization of VMCs in the hydrogel system is a complex process, further studies are required to understand and controllably regulate through combinational consideration of the functional properties of HA materials, VMC behaviors and reaction–diffusion process of morphogens, so that the self-organization of VMCs could be rationally controlled for generating artificial tissues with desired inner microstructures.

## Conclusion

Diverse types of self-organized 3D multicellular structures can be developed by applying Noggin and BMP2 in the VMC-HA hydrogel network. The engineered ECM constructed by modified HA hydrogel and exogenous cytokines could serve as a flexible platform for studying cellular behaviors such as spreading, migration, and proliferation during their self-organization. The observed morphologies of 3D multicellular architectures are most likely associated with Turing instability-controlled reaction–diffusion processes of morphogens, since the theoretical model can predict the experimental morphologies well. These multicellular structures could mimic the morphogenesis of trabecular bone and bone nodule formation. Our work provides a reliable and rational method for constructing 3D multicellular structures through the process of VMC self-organization.

## Additional files

Additional file 1: Supplementary Methods for the 3D selective Plane Illumination Microscopy (SPIM) imaging, clustered encapsulation of cells in 3D HA-hydrogels, alkaline phosphatase staining, and cell proliferation measurement. The test results for cellular migration in HA-hydrogel treated by exogenous proteins are also described. (DOC 4182 kb)

Additional file 2: Supplementary Figures S1-S7. **Figure S1** Schematic view of the SPIM imaging process. **Figure S2** Variation of morphology of the VMC culture under different experimental conditions. **Figure S3** Variation of morphology of the VMC culture over time. **Figure S4** The effect of Noggin on the local aggregation of VMC culture. **Figure S5** The effect of BMP2 (0.5ug/ml) on the spheroid formation of VMC culture. **Figure S6** Cellular migration test for VMC culture treated by exogenous proteins. **Figure S7** Histochemical demonstration of alkaline phosphatase activity in VMCs. (DOC 4145 kb)
