# Supplementary material for: Directing three-dimensional multicellular morphogenesis by self-organization of vascular mesenchymal cells in hyaluronic acid hydrogels
Source: J Biol Eng. 2017 Apr 3;11:12. doi: 10.1186/s13036-017-0055-6 (PMC5376694; doi:10.1186/s13036-017-0055-6)
Supplement: Supplementary file 1 — Supplementary Methods for the 3D selective Plane Illumination Microscopy (SPIM) imaging, clustered encapsulation of cells in 3D HA-hydrogels, alkaline phosphatase staining, and cell proliferation measurement. The test results for cellular migration in HA-hydrogel treated by exogenous proteins are also described. (DOC 4182 kb) [file 13036_2017_55_MOESM1_ESM.doc]

# Additional file 1 for

Directing Three-dimensional Multicellular Morphogenesis by Self-organization of Vascular Mesenchymal Cells in Hyaluronic Acid Hydrogels

Xiaolu Zhu^a,b^*, Shiva Gojgini^c^, Ting-Hsuan Chen^d^, Peng Fei^b^, Siyan Dong^b^,

Chih-Ming Ho^b,e^ and Tatiana Segura^c^*

**^a^** College of Mechanical and Electrical Engineering, Hohai University, Changzhou, Jiangsu 213022, China

**^b^** Mechanical and Aerospace Engineering Department, University of California, Los Angeles, Los Angeles, CA90095, USA

**^c^** Chemical and Biomolecular Engineering Department, University of California, Los Angeles, Los Angeles, CA90095, USA

**^d^** Department of Mechanical and Biomedical Engineering, City University of Hong Kong, Hong Kong, China

**^e^** Bioengineering Department, University of California Los Angeles, Los Angeles, CA 90095, USA.

*Correspondence should be addressed to: Xiaolu Zhu (zhuxiaolu@hhu.edu.cn, Tel: +86 519 85191840) and Tatiana Segura (tsegura@ucla.edu, Tel: +1 310 2063980)

# Additional file 1:

# Supplementary Methods

## *Selective Plane Illumination Microscopy (SPIM) for Three Dimentional Imaging.*

Selective Plane Illumination Microscopy (SPIM) is a first-of-its-kind technique that uniquely allows high resolution, isotropic imaging over a wide scale from microns to millimeters , which is especially useful for three dimensional imaging on large, thick fluorescent biological specimens. In our 3D tissue culture experiment, SPIM was utilized to visualize the multicellular morphology from micron-scale (single-cell) to millimeter-scale (multi-cellular) three-dimensionally. In contrast with conventional wide field microscopy and laser scanning microscopy, SPIM applies two separate sets of lenses for illumination and detection respectively . In a simplified SPIM setup, a low NA objective lens is combined with a cylindrical lens to generate a light blade for optically sectioning the sample transversely. As a result, the axial resolution of the system is determined by illumination. At the same time, by the use of low magnification lens, a large field of view (FOV) can be easily achieved without apparently sacrificing axial resolution. The sample container should be as least has two perpendicular sides, which are transparent and flat to let the light go through with minimum refraction and scattering.

***Clustered encapsulation of cells in 3D HA-hydrogels***

Clustered encapsulation of cells was made for studying cellular migration in HA-hydrogels. Cell clusters in fibrin gel clots were made by resuspending 300,000 VMCs in 10 μL of fibrin and thrombin solution (2 mg/mL fibrinogen and 2 U/mL thrombin). Then, the clusters of cells were made by dropping the suspension onto a sigmacoted plate and incubating at 37 C for 20 min. The clusters of cells inside fibrin clots were transferred into the HA-hydrogels by placing it inside the gel precursor solution. The gel was swelled in DMEM and cultured in DMEM supplemented with 15% FBS and 1% P/S.

## *Alkaline Phosphatase Staining.*

The color reaction solution was fresh prepared by adding 200 μL NBT/BCIP stock solution (Roche Diagnostics, IN) to 10ml of 1X detection buffer which is a mixture of 1ml of 1M Tris-HCl, 1ml of 1M NaCl solution, 0.5 ml of 1M MgCl_2_ solution and 7.5 mL distilled water. The sample was rinsed for 2 times with 2 mM Tris-HCl solution (pH 9.5). Then the sample was incubated in color reaction solution in the incubator until the development of color was observed (around 2 h). When the color had developed, the color reaction solution was removed from the sample to stop the reaction. Afterwards, the sample was washed twice in 2 mM Tris-HCl solution. Finally, the sample was fixed with 4% PFA at room temperature. The alkaline phosphatase activity was served as an indicator for the differentiation of the VMCs into bone cells .

## *Cell proliferation measurement*

The AlamarBlue (Life Technologies, Grand Island, NY, USA) assay was used to quantify the relative metabolic activity of the cells inside the hydrogels, which could be used to measure the cell proliferation over time. In this assay, 15 μL of AlamarBlue dye was mixed with 100 μL phenol red free DMEM and added to each gel-containing well in the 96 well plates and incubated at 37 °C with 5% CO_2_ for 4 h. After incubation with the reagent, the fluorescence signal was quantified using a plate reader with an excitation and an emission wavelengths of 550 nm and 590 nm, respectively. The proliferation rates were presented as fold increase over the value obtained on the first day. Acellular hydrogels were used to adjust for background fluorescence.

# Supplementary Results

## *Test results for Cellular migration in HA-hydrogel treated by exogenous proteins*

The migration rate of cells was determined by the net result of the cellular diffusion and chemotactic effect that counteract each other for cellular motions. In the simulation model, the cellular diffusion of cells could be enhanced by increasing the value of *q*; the chemotactic motion of cells could be enhanced by increasing the value of *χ*. According to the migration tests for cell clusters inside HA-hydrogels with the above experimental conditions (see Fig. S6), the averaged migration rate of the cells in Noggin-treated sample was larger than that in the control one (refer to the slopes of the linear fitting in Fig. S6j-k). Thus, *q* = 0.006 for noggin treated sample was estimated compared to *q* = 0.004 for the control, since larger *q* leads to a quicker migration away from an aggregated cellular cluster. On the other hand, the experimentally measured migration rate of cells in BMP-2 treated sample was averagely the smallest among the three cases in Fig. S6 (refer to the slopes of the linear fitting in Fig. S6j-l). Thus, the increased *χ* = 0.10 for BMP-2 treated sample was set compared to the control, since larger *χ* value makes the cluster of cells more aggregated, which leads to a slower migration away from the cellular cluster. In contrast, the $V_{EX}$ was set as positive value (8×10^-6^) when exogenous BMP-2 was applied. All other parameter values are the same among these three cases. In summary, the mathematical model based on Turing instability can well model the variations of the 3D multicellular architectures when different exogenous factors are applied.

References

1. Verveer PJ, et al. High-resolution three-dimensional imaging of large specimens with light sheet-based microscopy*.* Nat Methods. 2007; 4(4): 311-313.

2. Huisken J, et al. Optical sectioning deep inside live embryos by selective plane illumination microscopy*.* Science. 2004; 305(5686): 1007-1009.

3. Kilian KA, et al. Geometric cues for directing the differentiation of mesenchymal stem cells*.* Proc Natl Acad Sci USA. 2010; 107(11): 4872-4877.
