# Supplementary material for: Directing three-dimensional multicellular morphogenesis by self-organization of vascular mesenchymal cells in hyaluronic acid hydrogels
Source: J Biol Eng. 2017 Apr 3;11:12. doi: 10.1186/s13036-017-0055-6 (PMC5376694; doi:10.1186/s13036-017-0055-6)
Supplement: Supplementary file 2 — Figure S1 Schematic view of the SPIM imaging process. Figure S2 Variation of morphology of the VMC culture under different experimental conditions. Figure S3 Variation of morphology of the VMC culture over time. Figure S4 The effect of Noggin on the local aggregation of VMC culture. Figure S5 The effect of BMP2 (0.5ug/ml) on the spheroid formation of VMC culture. Figure S6 Cellular migration test for VMC culture treated by exogenous proteins. Figure S7 Histochemical demonstration of alkaline phosphatase activity in VMCs. (DOC 4145 kb) [file 13036_2017_55_MOESM2_ESM.doc]

# Additional file 2 for

Directing Three-dimensional Multicellular Morphogenesis by Self-organization of Vascular Mesenchymal Cells in Hyaluronic Acid Hydrogels

Xiaolu Zhu^a,b^*, Shiva Gojgini^c^, Ting-Hsuan Chen^d^, Peng Fei^b^, Siyan Dong^b^,

Chih-Ming Ho^b,e^ and Tatiana Segura^c^*

**^a^** College of Mechanical and Electrical Engineering, Hohai University, Changzhou, Jiangsu 213022, China

**^b^** Mechanical and Aerospace Engineering Department, University of California, Los Angeles, Los Angeles, CA90095, USA

**^c^** Chemical and Biomolecular Engineering Department, University of California, Los Angeles, Los Angeles, CA90095, USA

**^d^** Department of Mechanical and Biomedical Engineering, City University of Hong Kong, Hong Kong, China

**^e^** Bioengineering Department, University of California Los Angeles, Los Angeles, CA 90095, USA.

*Correspondence should be addressed to: Xiaolu Zhu (zhuxiaolu@hhu.edu.cn, Tel: +86 519 85191840) and Tatiana Segura (tsegura@ucla.edu, Tel: +1 310 2063980)

# Supplementary Figures


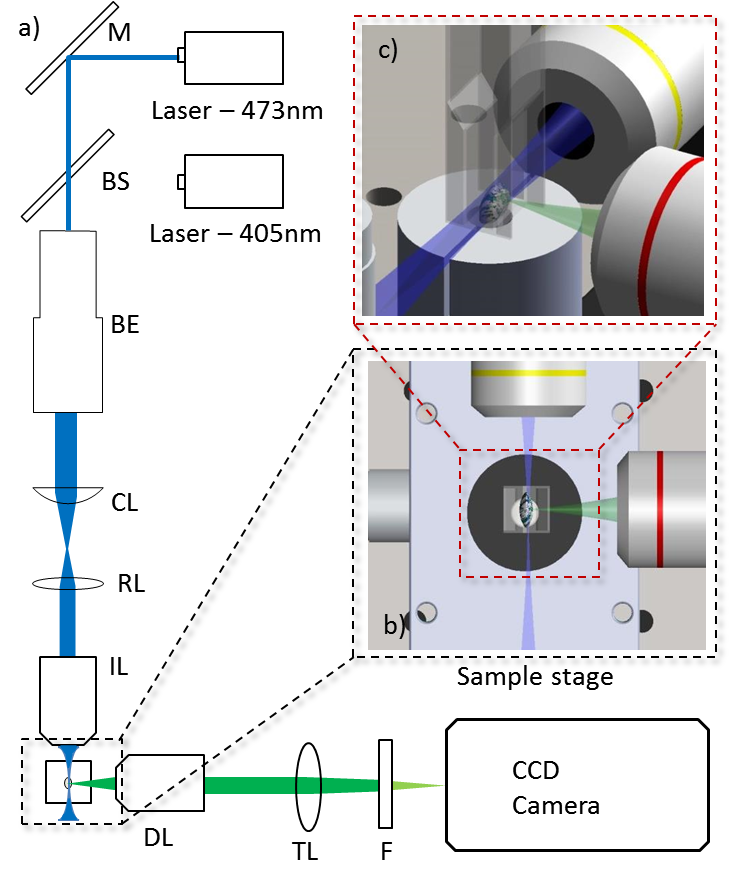


**Supplementary Figure 1** Schematic view of the SPIM imaging process. (a) Optical setup of the SPIM system. Collimated lasers (405 nm and 473 nm) were precisely aligned, fused, and transmitted through a lens set (CL, RL and IO), to yield alight sheet across the sample. The detection arm including a 4x or 10x objective lens (DO) was placed orthogonal to the illumination path for fluorescence detection. M, mirror; BS, beam splitter; BE, beam expander; CL, cylindrical lens; RL, relay lens; IO, illumination objective; DO, detection objective; TL, tube lens; F, filter. (b) and (c), Close-up view of the imaging strategy. The IO, DO and the samples were carefully aligned to make (1) the waist of the light sheet in line with the detection axis; (2) the light sheet illuminated plane exactly conjugated with the CCD plane. The optical cuvette containing the sample was mounted on a three-axis XYZ translation stage, which allowed rapid raster scan of the sample. During image acquisition, the sample was excited and scanned by the laser sheet along the Z direction. Accordingly, the CCD camera yielded a sectional image sequence, which contains abundant information deep inside the 3D sample. The image acquisition was implemented using customized LabVIEW program.

**Supplementary Figure 2 Variation of morphology of the VMC culture under different experimental conditions.** (a-b) Cell morphology on day 4 at different RGD concentrations (3.3% HA, r = 0.35). (c) Proliferation of cells over time at different RGD concentrations (3.3% HA, r = 0.35). (d-e) Cell morphology on day 5 at different cross-linker concentrations characterized by the parameter *r* (3.0% HA, 150 µM RGD). (f) Cell proliferation over time at different *r* values (3.0 % HA, 150 µM RGD).


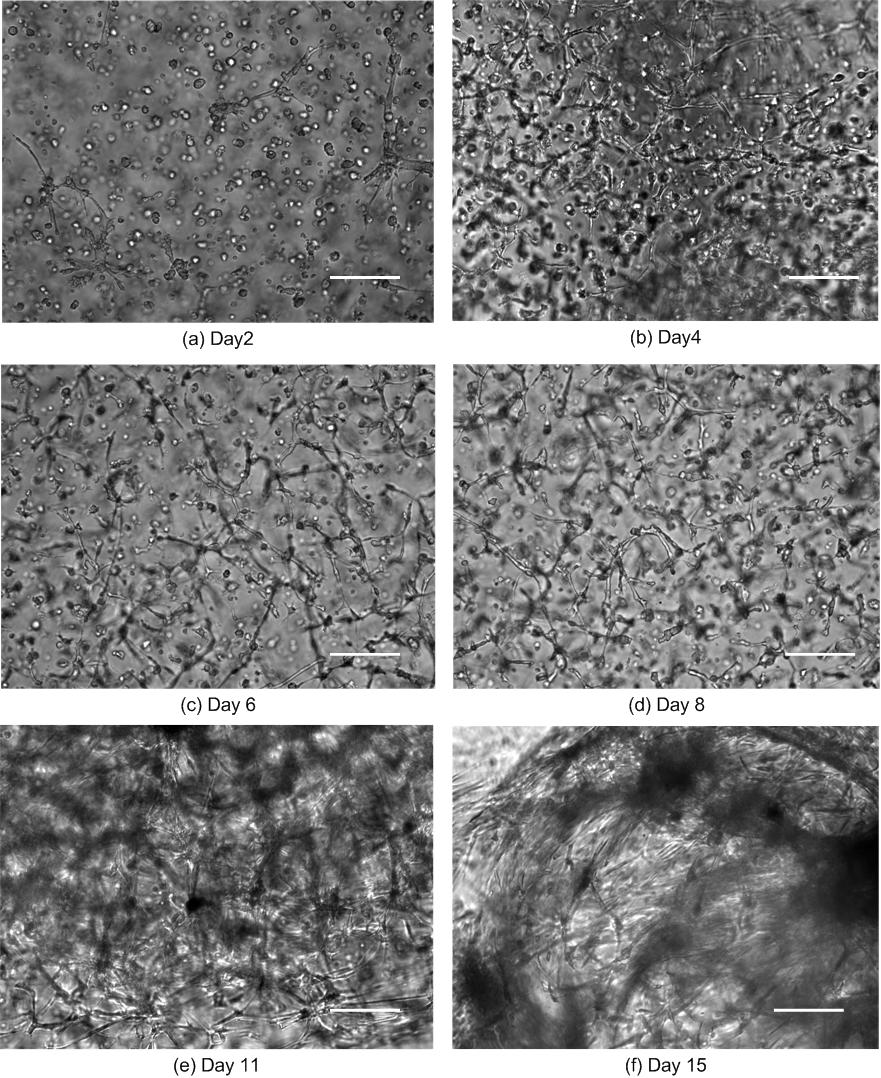


**Supplementary Figure 3 Variation of morphology of the VMC culture over time.** (a) Cell started to spread and sprout on day 2. (b) More cells sprouted and connected to each other on day 4. (c-d) The network morphology was persisted in the following several days. (e) Local cells aggregated into a thicker network structures on day 11. (f) More cells aggregated and connected with each other and finally formed larger dense blocky structures on day 15. All the scale bars are 200 μm.


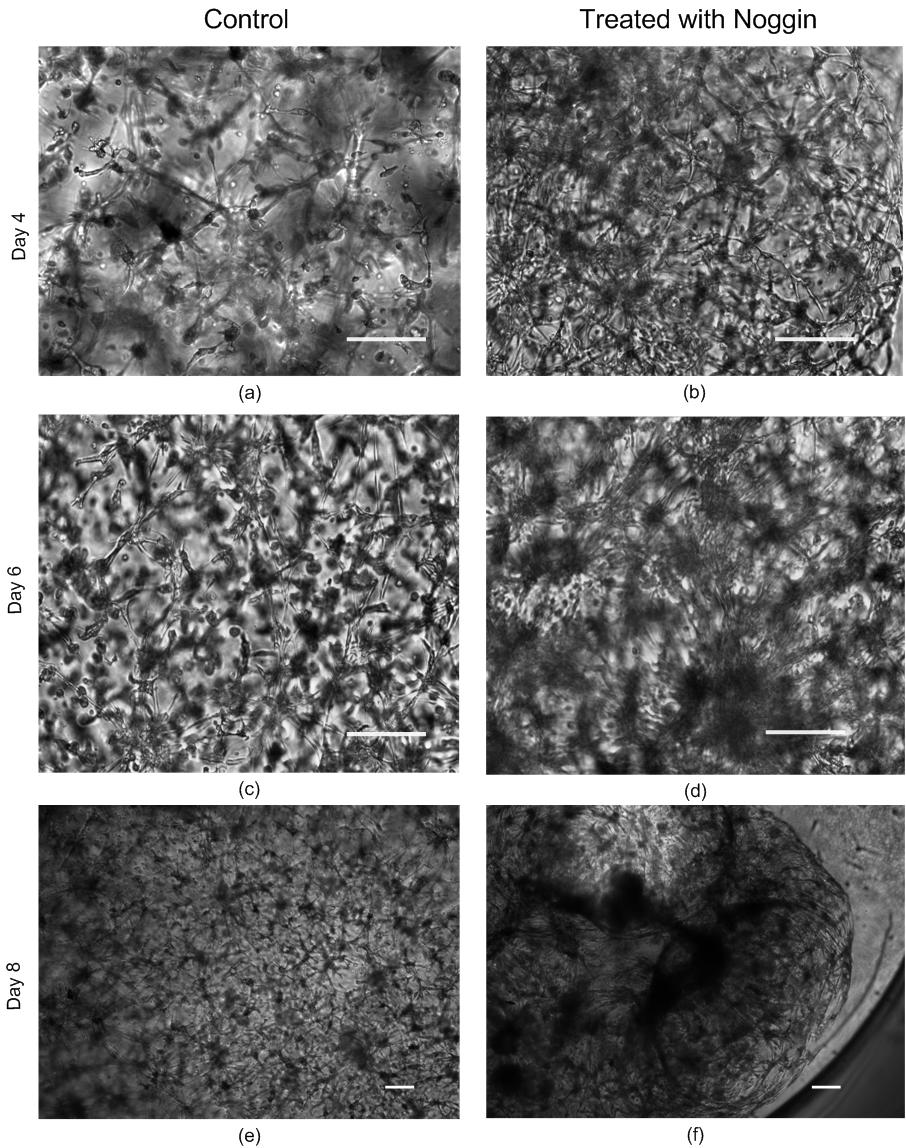


**Supplementary Figure** **4** **The effect of Noggin on the local aggregation of VMC culture**. The initial cell density for the sample was 7500/μL. The culture media containing noggin (0.25 μg/mL) was applied on day 0 and day 1. Noggin concentration of 0.5 μg/mL was applied from day 2 to day4. Noggin concentration of 0.9 μg/mL was applied from day 5 to day 9. All the scale bars are 200 μm.

**
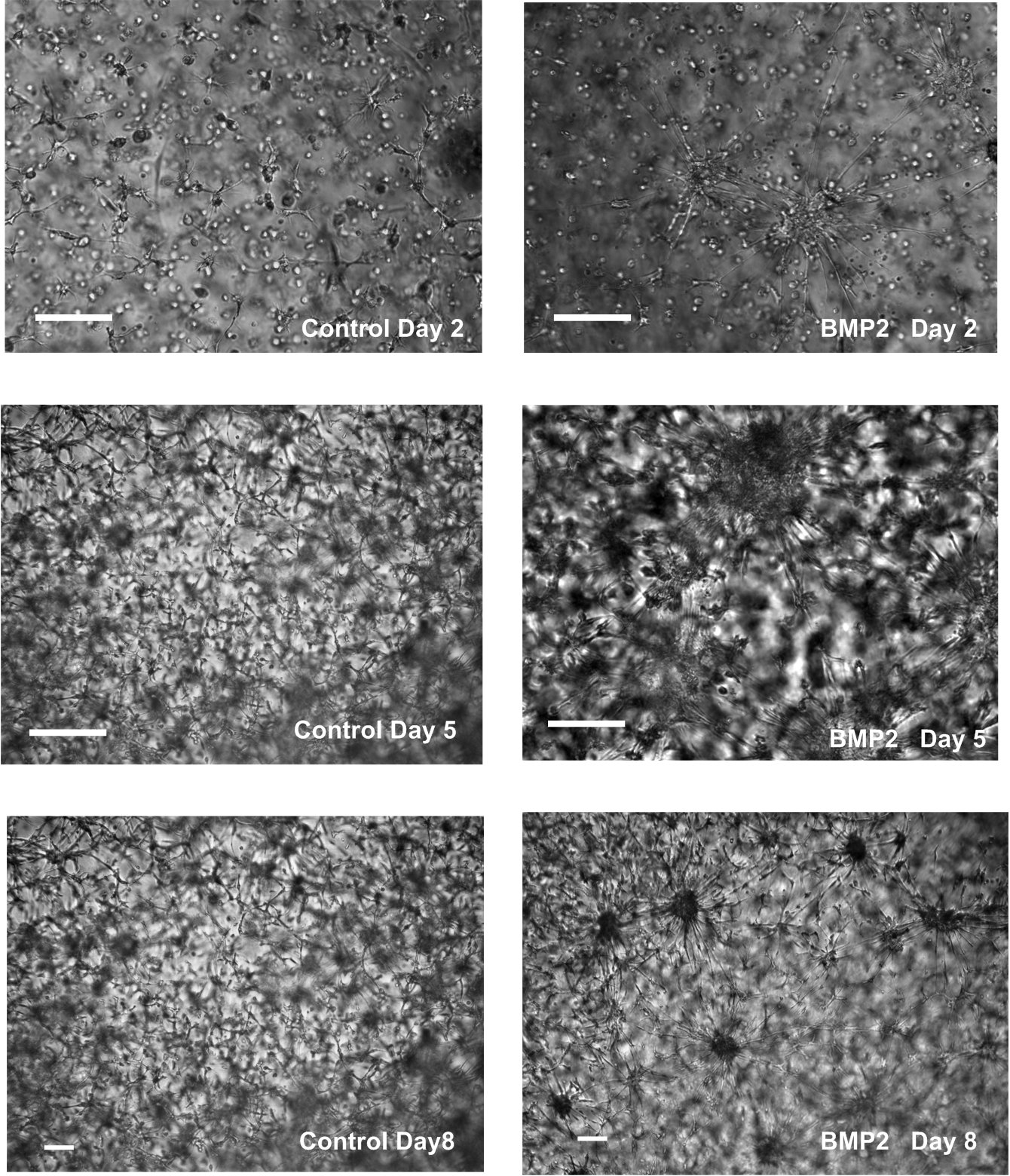
**

**Supplementary Figure 5 The effect of BMP2 (0.5 µg/ml) on the spheroid formation of VMC culture.** All the scale bars are 200 μm.


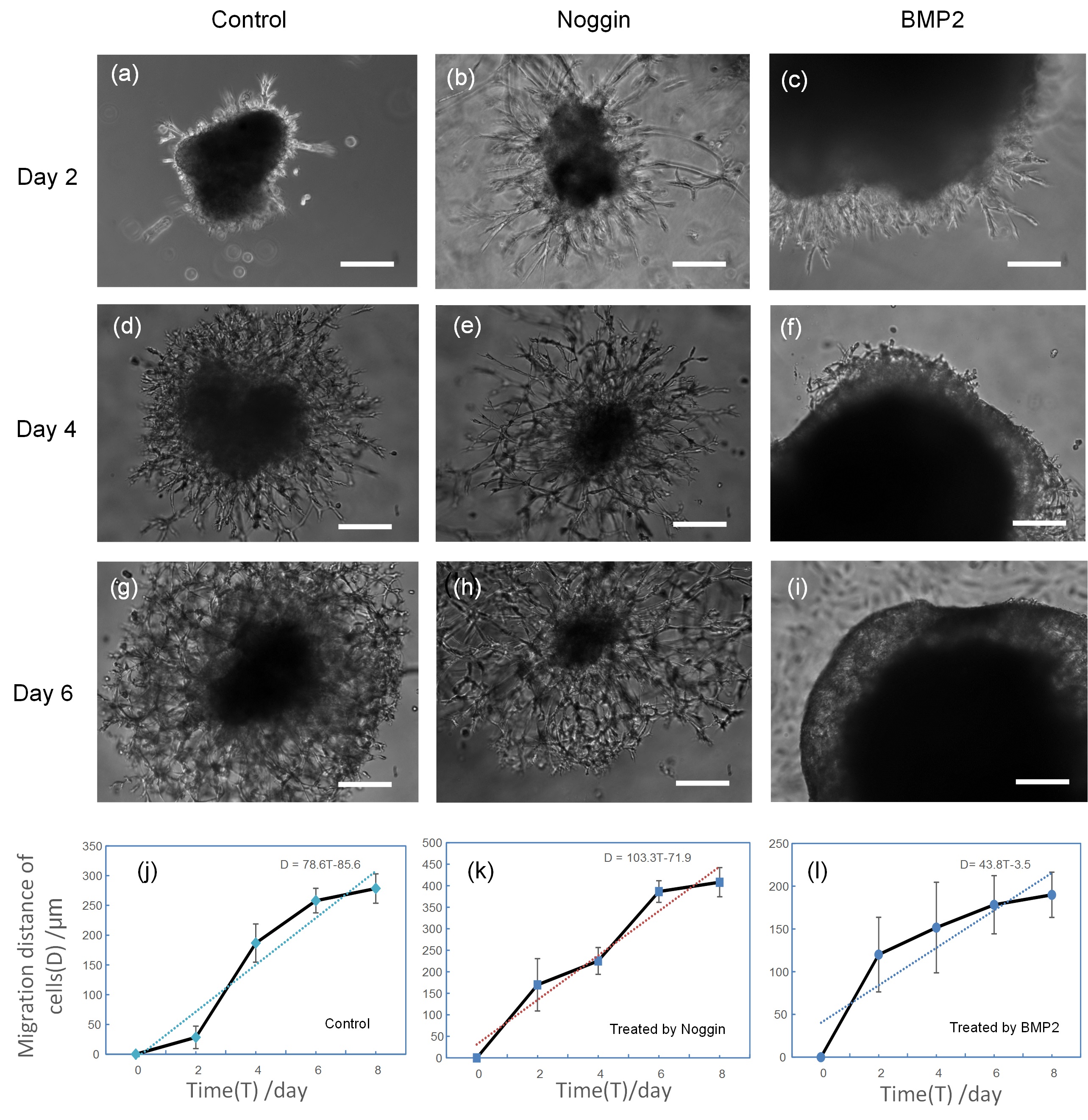


**Supplementary Figure 6 Cellular migration test for VMC culture treated by exogenous proteins.** (a-i) showed the morphologies of the cellular migration. Noggin made the cells migrate out of the cellular cluster in fibrin clot more quickly than the control (j, k). BMP2 made the cells migrate out of the clot a bit quicker with first 2 days than control but conspicuously slower than the control after day 2 (j, l). After day 3, the VMC culture treated by BMP2 had a slower migration rate than both the control and Noggin-treated VMCs (j-l). Conditions: 3.5% HA, 100 μM RGD, *r* = 0.6. All the scale bars are 200 μm.


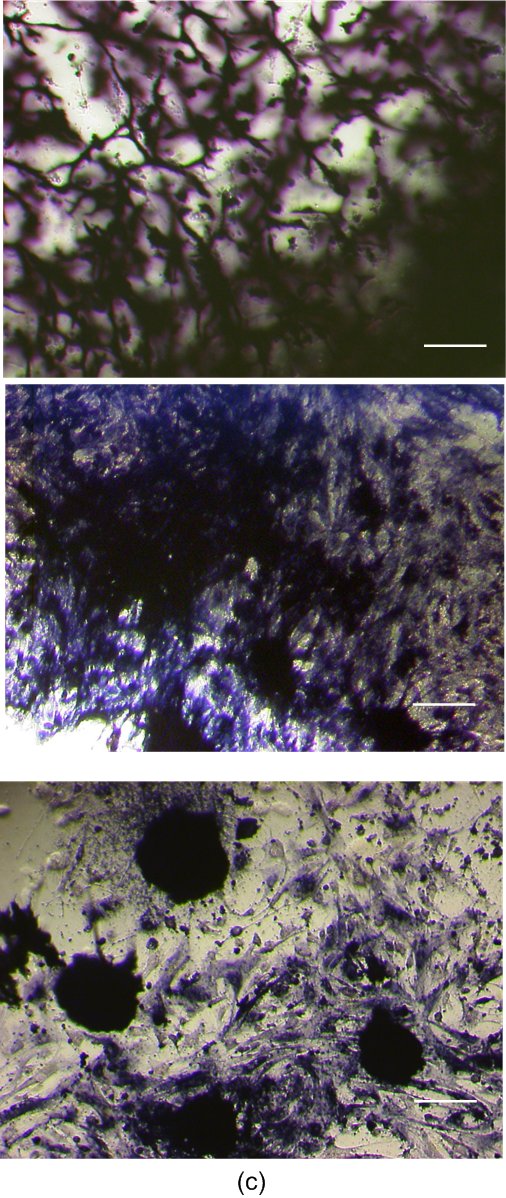


**Supplementary Figure 7 Histochemical demonstration of** **alkaline phosphatase activity in VMCs without treatment (a), with Noggin treatment (b) and with BMP2 treatment (c)**. All the scale bars are 200 μm. The darker area indicated the higher level of the alkaline phosphatase activity, which demonstrated that the VMCs had been differentiated into the bone cells (osteoblasts).

References

1. Verveer PJ, et al. High-resolution three-dimensional imaging of large specimens with light sheet-based microscopy*.* Nat Methods. 2007; 4(4): 311-313.

2. Huisken J, et al. Optical sectioning deep inside live embryos by selective plane illumination microscopy*.* Science. 2004; 305(5686): 1007-1009.

3. Kilian KA, et al. Geometric cues for directing the differentiation of mesenchymal stem cells*.* Proc Natl Acad Sci USA. 2010; 107(11): 4872-4877.
